# 

**Published:** 1874-04

**Authors:** 


### Books and Pamphlets Received.

A Treatise on Pharmacy; Designed as a Text Book for the student, and as a Guide for the Physician and Pharmacist. Containing the officinal and many unofficinal formulas, etc. By Edward Parrish, late Prof. of the Theory and Practice of Pharmacy, in the Philadelphia College of Pharmacy. Fourth Edition, revised and enlarged. By Thos. S. Wegand. Philad lphia: Henry C. Lea, 1874. Buffalo: T. Butler & Son.

The Puerperal Diseases. Clinical Lectures delivered at Bellevue Hospital. By Fordyce Barker, M. D. New York: D. Appleton & Co., 1874. Buffalo: Martin Taylor.

An Introduction to Physical Measurements, with Appendices on Absolute Electrical Measurement, etc. By Dr. F. Kohlrausch. Translated from the Second German Edition. By Thos. H. Waller, B. A., B. Sc. and Henry R. Proctor, F. C. S. New York: D. Appleton & Co., 1874. Buffalo: Martin Taylor.

Treatment of Nervous and Rheumatic Affections. By Static Electricity. By Dr. A. Arthius. Translated from the French. By J. H. Etheridge, M. D. Chicago: W. B. Keen, Cooke & Co., 1874.

Origin on Metalliferous Deposits. By Prof. T. Sterry Hunt. The Phenomena of Sleep. By Dr. Richardson. No. 10 of Half Hour Recreations in Popular Science. Boston: Estes & Lauriat. Buffalo: H. H. Otis.

Insects of the Garden; Their Habits, &c. By A. S. Packard, Jr. No. 2, of Half-Hour Recreation in Natural History.

A Manual of Toxicology, including the consideration of the Nature, Properties, Effects and Means of Detection of Poisions, more especially in their Medico-Legal Relations. By John J. Reese, M. D. Philadelphia: J. B. Lippincott & Co., 1874. Bnffalo: Martin Taylor.

Ligation of Arteries. By Dr. L. H. Farabeuf. Translated by John D. Jackson, M. D. Illustrated. Philadelphia: J. B. Lippencott & Co., 18 4. Buffalo: Martin Taylor.

### Medical and Surgical Journal

PUBLISHED MONTHLY,

Containing Original Articles, Reports of Medical Societies and Hospitals. Editorial, Reviews, Correspondence, Army News, etc., etc; including the usual variety of Medical Periodical Publications. Specimen copies sent on application.

Address Buffalo Medical & Surgical Journal, Buffalo, N. Y.

TERMS OF SUBSCRIPTION, - - $3.00 PER YEAR, IN ADVANCE.

